# Health and Disease

**Published:** 1861-01

**Authors:** 


### HALL'S JOURNAL OF HEALTH.

**Our Legitimate Scope is almost boundless: for whatever begets pleasurable and harmless feelings, promotes Health; and whatever induces disagreeable sensations, engenders Disease.**

WE AIM TO SHOW HOW DISEASE MAY BE AVOIDED, AND THAT IT IS BEST, WHEN SICKNESS COMES, TO TAKE NO MEDICINE WITHOUT CONSULTING A PHYSICIAN.

**Vol. VIII.] JANUARY, 1861. [No. 1.**

### HEALTH AND DISEASE.

Two months ago, one of the very sweetest "teens" of the sunny South possessed herself of our book on *Health and Disease*, as she was about to return to her home of sunshine and of flowers, and writes of it to a New-York lady: "Dr. Hall's book was my constant companion on the steamer. I studied it all day, and slept with it under my pillow at night." A happy thing would it be for the daughters of our land, if such a book could replace the blood-and-murder newspaper, the trashy monthly, the infidel magazine, and the corrupting novel; a book which does not deal in symptoms, which never advises a dose of medicine, and rides no hobby, but which seeks to show how disease is engendered, and how health may be maintained, the happiness of being always well, and the calamity of being always sick. Instructions such as these are absolutely essential to the well-being of the young, many of whom spend a large portion of their time in fictitious reading, yet give not an hour in a day, a week, a month! to the study of the preservation of that health which is the foundation and conservator of personal beauty and social and domestic enjoyment. We earnestly invite every subscriber who has children, to present a copy of the book to each one of them, with the injunction to read it once a year carefully and deliberately, for many a household is made childless prematurely, in consequence of ignorance of two or three general principles laid down therein with the greatest clearness.

But how little did that sweet girl think while she was penning her letter, that a portion of it would appear in print, and before the close of the year, would be found in a bound volume, in the largest libraries, North and South, East and West, and that she was contributing her mite to the sum total of the enjoyment of many thousands of readers. Happy he who wins such a prize; but he will have to fight for it, as it seems there is a cordon of black hearts! around it, ready to resist the capture of "young missus," with all the desperation of a simple and pure affection; for read a little further: "We did not get any sleep on the steamer, and retired immediately on our arrival home, but could not possibly get any repose, for as soon as the negroes heard that we had retired, they must all come to see us, and if they found us asleep, they would give us such a shaking and hugging, (some body would have liked to have been a nigger just then, for a spell any how,) that I was glad enough to get up and keep my face out of their reach, and receive all their demonstrations on my neck and arms. They would hold us out at arms' length, and comment quite amusingly on our appearance. Cumseh and Corah were wild with delight—they did nothing but grin and run after us for two days." And then she goes on to distribute the presents among the little curly-heads. There is moral beauty in scenes like these; they remind us of the by-gones of our own sunnier childhood; and one other that we often think of in these frigid climes, and which might bear repetition in every Northern family where there are servants. But how many Northern families take that interest in the soul's welfare of their hired help? Does one family in ten do it? or in one thousand? We see grandmother now, with her jolly fat cheeks, united with puritanic determination, seated in a chair, the Shorter Catechism in one hand and Watts' divine and moral songs in the other; the little negroes standing around in a huge semicircle, all washed and clean, after breakfast; the arms and hands of each making a pyramid, the apex of which, formed by the two fore-fingers of each hand, touched the "septum," the partition of the nostril, for they were going to "say their prayers." How their little black eyes twinkled! A few were sedate; some assumed a mock solemnity; but the greater number gave too clear proof of *their* total depravity, in the sly twinkle, or "irrepressible conflict," not Gov. Seward's, but the vain battle between a solemn look and a titter. The solemns, however, almost always came out second best, and there was a general explosion and utter demoralization of the ranks! and grandmother would let her hands fall down helplessly in her lap and sigh for their degeneracy. But like her grandson, she never gave up any thing, and would always "put 'em through" the curriculum. But she and many of them have long since passed away, and no doubt now occupy the same platform in heaven where the vain distinctions of time and human weakness are never known.

But where have we wandered? We must confess that we are powerless to "make the connection" between teaching little negroes to say their prayers and the legitimate objects of a Journal of Health, except it be in the direction that if every Christian family would strive to do its whole duty towards domestics, as did our grandmother, in taking a deep, sincere, and pious interest in their religious welfare, there would at once be swept from our households a very large share of the causes of disquietude, of fretfulness, and almost hourly irritation, found in unfaithful, incompetent and unprincipled servants, and which have so large a share, in the long run, in eating out that gladness of heart and joyousness of spirit, the absence of which is so fruitful of both bodily and mental maladies. But alackaday! we fear the times are so much out of joint now, that multitudes of intelligent households are all guiltless, not only of personal religious teaching of the servants, but of the children too, their time being taken up in reading "moral" magazines and books crammed full of "religious fictions," as a compromise on "Sunday reading" between the Bible and fashion-plate monthlies. Let each mother make a note of it, and put it down in parallel columns, how much time of each day for any week is expended in discussing and deciding the points as to the cost and style and tint of the various articles of dress, how much in "fixing" for "the party," for the dancing-school, the "receptions" and the "at-home" days, and then how much is devoted to loving teachings at the knee, about the vanities of time, the shortness of life, the certainty of death, and the means of preparation therefor. Then let another parallel column be made, let one state the sum total of efforts made to teach our children how to live for themselves, and in the other how to live for others. The benevolent live long, healthfully, happily, and in honor; the selfish, the wicked, shall not live out half their days.

The above was crowded out of the December number aftei it was already in type. That ignoble quality, worldly policy, would have given it, to the exclusion of any other article, as all subscriptions ended with that number, and nearly half our patrons live south of Mason and Dixon's line. It might have tempted some to renew, who, without it, would not. But we don't choose to live either by politics or policy; both debase.

